# Aiming for better use of convenience food: an analysis based on meal production functions at home

**DOI:** 10.1186/s41043-020-0211-3

**Published:** 2020-02-11

**Authors:** Satoshi Nakano, Ayu Washizu

**Affiliations:** 1 The Japan Institute for Labour Policy and Training, 4-8-23, Kamishakujii, Nerima-ku, Tokyo, 177-8502 Japan; 2 Waseda University, 1-6-1, Nishiwaseda Shinjyuku-ku, Tokyo, 169-8050 Japan

**Keywords:** Home production, Indicator of cooking effort, Indicator of convenience food usage intensity, Smart food system, Proximity score

## Abstract

**Background:**

In recent years, the evaluation of convenience food has changed. It came to be considered not to have a negative effect on health and is now positioned as a tool to support dietary habits of elderly and other people. In advanced countries where the population is aging, convenience foods are expected to improve the eating habits of the elderly.

**Methods:**

We defined the indicators of cooking effort and usage intensity of convenience food and presented a model wherein a “meal” is home-produced. In the model, a home cook decides the optimal cooking effort to apply for a given usage intensity of convenience food. Using an empirical form of the proposed model, we performed a multiple regression analysis and calculated “the elasticity of cooking effort with respect to the usage intensity of convenience food” for home cooks, with each attribute defined by a combination of different personality and demographic factors, using the estimated coefficients.

**Results:**

Regression analysis results revealed a negative correlation between cooking effort and the usage intensity of convenience food, which is consistent with our theoretical model of home meal production. The results showed that home cooks who have special food preferences may not be satisfied with accepting convenience foods purchased from the market as they are and that these home cooks will require a higher cooking effort to obtain higher satisfaction. The elasticity of elderly home cooks was low, implying that they are not flexible enough to accept convenience food.

**Conclusions:**

The results revealed that existing convenience foods do not have the same impact on home cooks with attributes. This problem can be solved with smart food systems that utilize information and communication technology, which allow home cooks to explore information on convenience foods that match their preferences and enable food providers to offer food that matches the specific tastes of home cooks. The regression results suggest this possibility.

## Background

Can convenient foods contribute to improving the quality of home diets? According to previous research related to people’s demand for convenience in food preparation, Bava et al. [1] concluded that people need convenience in food preparation to reduce time and cognitive effort. However, Contini et al. [2] pointed out that people fear negative judgment from close friends and relatives as a result of their choosing convenience food. In addition, it has been pointed out that diet quality is degraded, and health is adversely affected if convenience food is used to save time for food preparation [3–5]. The same is true for middle-income families in the Asia-Pacific region in recent years [6]. Veflen Olsen et al .[7] discussed how consumers would choose a diet that is both convenient and healthy. Stranieri et al. [8] also pointed out that convenience food has a negative environmental impact and examined the factors affecting the consumers’ acceptance of healthy and environment-friendly convenience food.

Despite the negative impact of using convenience food to save food preparation time, Adams and White [9] pointed out that convenience food is overstated as a factor contributing to poor health. Furthermore, it seems that various factors associated with personal values for eating habits influence people on how to spend time on food preparation or using convenience food [10–12]. Convenience foods have various benefits beyond saving time [13]. Through researching the factors that affect the demand for convenience food, an idea has been constructed that the use of convenience foods does not fundamentally change the diets of people but assists them. For example, convenience foods would assist people in single households, the elderly, and those with low cooking skills [14–16]. Jackson and Viehoff [17] attempted to review the meaning of convenience food in such a context. They regarded convenience food as a socially, economically, and culturally acceptable culinary innovation that is important for domestic routines. Further, against the background of a rapidly aging population in developed countries, several studies have pointed out that convenience food may improve eating habits and prevent malnutrition in the elderly [18–20]. A well-planned, ready-made meal will help elderly people who lack the physical strength for cooking and who tend to have a bias in food preferences. Well-managed meals maintain the health of the elderly, so assisting home cooks in making such meals is now a major policy issue in Japan [21–23].

According to the United Nations (UN) population data [24], Japan has the largest proportion of elderly among developed countries, and the diet of the elderly is becoming a major social issue. The Japanese government cited a smart food system as one of the goals of Society 5.0, which is one of the care measures of the aging population [25]. It is desirable that smart food systems using information and communication technology (ICT) provide the elderly with knowledge about eating habits and assist them in using convenience foods. Using a smartphone, elderly people can easily get information on food and nutrition that suits their tastes or can buy their favorite foods from distant stores over the Internet. Monteban et al. [26] have discussed the role of social connection and information exchange for healthy food access. In a smart society, such information networks will expand. ICT-embedded urban systems that use a digital information platform facilitate more efficient and effective urban management and realize high-quality human and social capital [27]. The elderly with cognitive impairments also perceive ICTs to be useful when they meet their needs in daily activities [28]. Furthermore, Nakano and Washizu [29] conducted an empirical analysis of food supply structure in such a smart food system and concluded that it creates a new economic cycle and business opportunities.

As mentioned above, in developed countries, there is currently a growing interest in various social roles of convenience foods. Convenience foods are considered to have a variety of benefits other than time savings. They assist with people’s eating habits and contribute to healthy eating habits of the elderly. In this study, we consider such convenience foods to be useful, especially in aging societies of advanced countries, and analyze the usage behavior of home cooks. The purpose of this study is, based on the theoretical considerations concerning the production and consumption of food at home, to build a model to analyze the home cook’s choice behavior related to convenience food and to use the model to quantitatively show the difference in the behavior of home cooks with various backgrounds. For our model construction, the recent report by Casini et al. [30] provides interesting suggestions. They defined convenience food as that which saves time and effort for cooking and then evaluated the preference for convenience food, i.e., willingness to pay for saving time spent on cooking by people with various backgrounds. They concluded that people’s time-saving behavior for cooking differs from that for other daily duties (such as commuting) and pointed out that there is no general tendency. Their analysis found segments such as those who value time saving in cooking and those who receive utility from cooking. Our model can explain the background of such segments. Furthermore, Lahne et al. [31] pointed out the importance of measurement tools for the research in consumer food behaviors. We have developed objective indicators that can be quantified for the concepts appearing in our model based on the results of a large-scale survey conducted in the Japanese metropolitan area.

Furthermore, our empirical results suggest that convenience foods may reduce cooking effort, but the effects were not uniform among home cooks with different attributes. It was found that elderly people, in particular, cannot effectively reduce cooking effort by using convenience food. Therefore, with our model, we examined the conditions necessary for such elderly people to be able to use convenience foods effectively and indicated that such conditions could be created under a smart food system that provides information appropriately using ICTs.

## Model

A theory on home production was developed by Becker [32] and pioneered by Gronau [33, 34]. As stated by Davis [35], the empirical home production model of food at home (FAH) is typically formulated as follows: consumers decide on the allocation of monetary spending on market goods and the time to prepare meals to optimize the combination of FAH and food away from home (FAFH). According to Davis, early studies focused on the opportunity cost of time to prepare a meal (usually in terms of market wages) as a determinant of FAH or FAFH demand. With the development of time-use surveys, the relationship between the opportunity cost of time and time allocation between FAH and FAFH started being studied. Since then, progress has been made in research dealing with the simultaneous allocation of market goods and time. Davis said that the share of time costs in FAH production, as well as the elasticity of substitution between market goods and time, brought important implications for food-related policies. Davis also argued that more work is needed to develop it into a non-unitary model, and some attempts have been made for that. For example, Raschke [36] measured shadow wages to make the value of domestic labor more realistic. There are also studies in which time is de-unitarized. Dunn [37] distinguished the time values of those who had retired and those who had not; Senia et al. [38] distinguished between the time of eating and food preparation; You and Davis [39] distinguished between the behavior of the children’s parents. Matsumoto [40] pointed out that the value of a spouse’s working time is related to the environmental behavior produced by a household, and You and Davis [41] considered the value of the spouse’s working hours in the evaluation of food benefits produced by a household. There were also some attempts to de-unitarize home food production theory for goods. Canelas et al. [42] classified inputs in home production functions into five categories and measured the elasticity of substitution between time and money for each category. Kohara and Kamiya [43] and Crossley and Lu [44] classified foodstuffs into those requiring time to prepare a meal and those not requiring it. These studies suggest that the choice of food is inseparable from the time needed to prepare the meal. As with Casini et al. [30], Crossley and Lu [44] also pointed out that the time spent on meals may be a period of enjoyment and may not necessarily be a constraint factor for utility maximization. Recent studies of Etile and Plessz [45] and Sharma et al. [46] have referred to innovations in cooking and food services as a shift factor in the production function of food. Davis and You [47] pointed out the need for human investment to remove time constraints, as these constraints are an obstacle to the implementation of nutrition policy targets.

We present below a home production model of meals, which adds the following points to the literature:

- Food was divided into 13 types according to the time required to cook it (degree of convenience).
- We proposed an indicator to measure the magnitude of cooking effort spent by home cooks who rarely enter the labor market, in preparing their meals (this indicator is similar to the time in previous research, but it has a concept that emphasizes “labor hour”). The budget constraint for home cooks is assumed to be exogenous since the other members of the household are the main earner.
- From the relationship between indicators of convenience and cooking effort under the condition of utility maximization, the elasticity of cooking effort for convenience of food was estimated (this elasticity is similar to that of substitution between food and cooking time in previous studies).
- From the estimation results, we considered implications for the development of new technology (“informatization” or “smartification”).

Our home production function for meal production at home is as follows. A meal (*M*) is produced at home by cooking effort (*E*) of the home cook:

1 $$ M=f(E), $$

subject to diminishing marginal productivity ${f}_E=\frac{\partial f}{\partial E}>0$ and ${f}_{EE}=\frac{\partial^2f}{\partial {E}^2}<0$. *M* is the measure of the physical or psychological richness of the table. A rich table is a variety of meals with a large number of dishes, or an elaborate meal created by a home cook with a great deal of effort. *E* represents not only the physical and mental effort but also the quantity of foodstuffs as ingredients. In other words, a great deal of effort is required to make the richness of the table.

Let *F* be the unit cognitive burden imposed by one unit of the home cook’s cooking effort. Home cooks were assumed to be altruistic with respect to the utility of household members, seeking to maximize the utility of the meal [48]. Gronau [34] used market wage as the unit burden imposed by labor input on the home production function. This is because the market wage is considered an opportunity cost that people give up when they use their labor for home production. However, uniform market wages are not applied to the cooking effort in the home meal production function. As Casini et al. [30] pointed out, the unit burden imposed by the cooking efforts of home cooks will be different for each home cook. We assume that the unit cognitive burden imposed by a home cook’s cooking effort can be properly measured by his/her usage intensity of convenience food. The intensity is assumed to be high when the home cook uses a lot of ready-made convenience food and low if he/she is cooking from scratch. If a home cook enjoys cooking, then the cognitive burden imposed by this cooking effort is small, and the usage intensity of convenience food will be low, and vice versa.

Then, the total cognitive burden imposed by the cooking effort spent by the home cook on his/her meal is represented by *F* ∙ *E*. Here, we assume a home cook behavior model in which the home cook maximizes the utility of the meal *u*(*E*) defined by the production of the meal minus the total cognitive burden imposed by the cooking effort:

2 $$ \underset{E}{\max }u(E)=f(E)-F\bullet E. $$

The first term on the right side of Eq. (2) indicates that a home cook’s cooking effort *E* increases the utility of his/her meal, while the second term shows that applying his/her cooking effort *E* increases his/her burden and lowers the utility of his/her meal. The maximization condition of Eq. (2) is as follows:

3 $$ {f}^{\prime }(E)=F $$

where *f*^′^(*E*) is the concept called marginal productivity of the cooking effort, which indicates the increment in meal produced by each additional unit of cooking effort. Under the maximization condition, this magnitude is compensated by the cognitive burden imposed by one unit of cooking effort. From the relationship in Eq. (3), it is helpful to empirically determine the magnitude of the change in cooking effort *E* with respect to the change in usage intensity of convenience food *F*.

As we do not have a priori information about the functional form of *f*^′^(*E*) in Eq. (3), by referring to Gronau [33], we formulated an explicit assumption about the functional form of *f*^′^(*E*). The function *f*^′^(*E*) is assumed to be linear:

4 $$ {f}^{\prime }(E)={\alpha}_0-{\alpha}_1E+{\boldsymbol{\alpha}}_{\mathbf{2}}\boldsymbol{y} $$

where ***y*** denotes the vector of variables affecting the marginal productivity of the cooking effort. Given this specific function and maximization condition (3), one can derive the cooking effort for home cooks by:

$$ E=\left({\alpha}_0-{f}^{\prime }(E)+{\boldsymbol{\alpha}}_{\mathbf{2}}\boldsymbol{y}\right)/{\alpha}_1, $$

5 $$ E={a}_0-{a}_1F+{\boldsymbol{a}}_{\mathbf{2}}\boldsymbol{y}. $$

Equation (5) indicates the optimal cooking effort *E* required for a given usage intensity of convenience food *F* for a home cook with particular attributes ***y***. Note that the estimates of coefficient −*a*_1_ is consistent with the theory should be negative. −*a*_1_ indicates the change in cooking effort for a given change in usage intensity of convenience food. In order to assess the importance of these changes to the current state, indicators of elasticity may be considered. An index of “the elasticity of cooking effort with respect to usage intensity of convenience food” indicating the percentage by which the cooking effort is reduced when the usage intensity of convenience food is increased by 1% is defined by the following equation:

6 $$ \varepsilon =-\frac{\frac{\partial E}{E}}{\frac{\partial F}{F}}=-\frac{\frac{\partial E}{\partial F}}{\frac{E}{F}} $$

Equation (6) can be estimated by dividing the estimated −*a*_1_ by the average observed $\frac{E}{F}$.

## Data and methods

### Data

Estimation of Eq. (5) in the previous section involves the use of a database named “Shokutaku (Table) Market Analysis and Planning (Shoku-MAP) [49]” provided by Lifescape Marketing Co., Ltd. Shoku-MAP is a database of daily purchasing and meal information of home cooks (400 households) who reside in the Tokyo metropolitan area (Tokyo, Kanagawa Prefecture, Chiba Prefecture, and Saitama Prefecture) with spouses, have families of two or more, and are 20–69 years old. The information is collected online. In addition to information about daily meals (dishes and foodstuffs) and purchasing (food commodity) information, awareness information about eating habits is collected. The data used in this study are daily meal data and awareness survey data for 2015 Shoku-MAP.

There are data on 79,444 home breakfasts (excluding eating out), 4706 home lunches, and 70,151 home dinners. This means that all meals that are either prepared at home or purchased outside and eaten at home are examined. Moreover, for each meal, dishes are classified into 978 items, and foodstuffs are classified into 2326 items. By collating dish data with foodstuff data, we can determine whether a home cook made his/her meal from scratch or used a ready-made meal.

Using this dataset, we created two kinds of indicators necessary for the empirical study of our model. The development of such indicators is a contribution of this research to the literature.

### Creation of indicators

#### Indicator for the cooking effort

The explained variable *E* in Eq. (5) is an indicator that represents the cooking effort. We assume that the cooking effort can be measured by both the number of dishes and the number of foodstuffs. The number of dishes will be proportional to the cooking time, and the number of foodstuffs will be related to the time to procure foodstuffs. Using the survey data in Shoku-MAP, we defined the indicator ${E}_{jn}^{dinner}$ of the *j*th person’s dinner *n* days after January 1, 2015, as follows:

7 $$ {E}_{jn}^{dinner}={Dish}_{jn}^{dinner}\bullet \sum \limits_i^{47}{Fnum}_{ijn}^{dinner} $$

$$ j=1,\cdots, 400;n=1,\cdots, 365 $$

Here, ${Dish}_{jn}^{dinner}$ represents the number of dishes in the *j*th person’s dinner *n* days after January 1, 2015; ${Fnum}_{ijn}^{dinner}$ represents the number of foodstuffs belonging to the *i*th sector of the input–output table in the *j*th person’s dinner *n* days after January 1, 2015. In Eq. (7), the indicator of cooking effort for each dinner is shown as the product of the number of dishes appearing on the dinner and the number of foodstuffs used to make them.

We have defined the same indicators as in Eq. (7) for breakfast and lunch.

#### An indicator of the usage intensity of convenience food

The explanatory variable *F* in Eq. (5) is an indicator that represents the usage intensity of convenience food. Using the survey data in Shoku-MAP, we defined the indicator ${F}_{jn}^{dinner}$ of the *j*th person’s dinner *n* days after January 1, 2015, as follows:

8 $$ {F}_{jn}^{dinner}=\sum \limits_i^{47}{Prox}_i\bullet {Sh}_{ijn}^{dinner} $$

$$ j=1,\cdots, 400;n=1,\cdots, 365 $$

Here, Prox_*i*_ represents the “proximity score” of the *i*th sector of the input–output table. ${Sh}_{ijn}^{dinner}$ represents the share of foodstuffs belonging to the *i*th sector of the input–output table in the *j*th person’s dinner *n* days after January 1, 2015. Equation (8) indicates the weighted average proximity score of the foodstuffs used in the dinner. Here, the “proximity score” is an index indicating the degree of food processing before the home cook obtains foodstuffs to be served. High proximity scores are assigned to processed foodstuffs that people can eat immediately, and low proximity scores are assigned to unprocessed foodstuffs (such as raw meat) that can only be eaten after cooking. “Convenience food” is considered to be a food with a relatively high proximity score. All foodstuffs are classified into the input–output table by the Ministry of Internal Affairs and Communications (MIC) of Japan, and a proximity score is assigned to each input–output classification as shown in Table 1. The classification of the input–output table is convenient for organizing the proximity score as it is categorized according to the production process. In Table 1, we give low proximity scores to food categories that require a lot of cooking effort to actually eat. For this reason, given the background of Japanese eating habits, meat with a low possibility of raw consumption is given a lower score than vegetables and seafood with a high possibility of raw consumption. We also give higher scores to food categories that are more likely to complete a meal. We believe that convenience foods replace the effort needed to make a meal, and the likelihood of completing the meal is also related to this score. Alcoholic drinks are given a lower score than other processed foods because they cannot complete a meal alone and still require other efforts.

**Table 1:** Proximity score of foodstuffs

Proximity score	Classification of input–output table that foodstuffs correspond to
1	111704* animal oil and fats, vegetable oil, and meal; 111705 condiments and seasonings; 111701 sugar; 111703 dextrose, syrup, and isomerized sugar; 202903 salt; 208909 miscellaneous final chemical products
2	111101 meat
3	011202 pulses, 011302 vegetables (under facilities), 011301 vegetables (outdoor), 011401 fruits, 011509 miscellaneous edible crops, 015301 special forest products (including hunting)
4	017101 marine fishery; 017102 marine aquaculture; 017201 inland water fishery; 017202 inland water fishery; 111309 miscellaneous processed seafood; 111301 frozen fish and shellfish; 111302 salted, dried, or smoked seafood; 012104 hen eggs
5	111401 grain milling
6	111501 noodles
7	112101 refined sake, 112109 miscellaneous liquors, 112102 malt liquors, 112902 soft drinks, 112903 manufactured ice, 112901 tea and roasted coffee
8	111503 confectionery
9	111201 processed meat products, 111203 dairy farm products, 111202 bottled or canned meat products, 111304 fish paste, 111303 bottled or canned seafood, 111602 preserved agricultural foodstuffs (except bottled or canned), 111601 bottled or canned vegetables and fruits, 111909 miscellaneous foods
10	111502 bread
11	111901 prepared frozen foods, 111902 retort foods
12	111903 dishes, sushi, and lunch boxes

*The six-digit number is the classification number of the input–output table by MIC, Japan

This indicator is larger when a small number of convenience foods are placed on the table than when cooking from scratch with several raw foodstuffs. We have defined the same indicators as in Eq. (8) for breakfast and lunch.

#### Numerical example of indicators

Table 2 demonstrates an application example of our indicators. Three types of meals with “katsu-don” as the main dish are shown in Table 2. Katsu-don is a bowl of rice topped with slices of deep-fried pork (Japanese style pork cutlet), beaten egg, and slices of onions cooked in a sweet soy sauce-based broth. It is relatively popular as a lunch or dinner dish and is a representative product of the “takeaway lunchbox” food market. At meal no. 1, takeaway lunchbox katsu-don purchased at the food market is served as it is bought. At meal no. 2, beer is served with the takeaway lunchbox katsu-don. Meal no. 3 assumes that the katsu-don is cooked from scratch at home. Meal no. 1 has a low effort indicator of 1 and a high convenience indicator of 12. However, at meal no. 2, if 1 item (beer) is added, the effort indicator increases to 4, and the convenience indicator decreases to 9. The effort indicator increases, since it is assumed that cooking effort will increase as the number of dishes and foodstuffs increases. For meal no. 3, in which the katsu-don was home-cooked, the effort indicator increases to 10 and the convenience indicator decreases significantly to 1.6. Our indicator seems to express a negative correlation between cooking effort and convenience food usage.

**Table 2:** Application example of our indicators

Meal no.	Menu contents	Indicator for usage intensity of convenience food (*F*)	Indicator for cooking efforts (*E*)
1	Takeaway lunchbox katsu-don	12	1(dish) × 1(foodstuff) = 1
2	Takeaway lunchbox katsu-don and beer	9	2(dish) × 2(foodstuff) = 4
3	Home-cooked katsu-don	1.6	1(dish) × 10(foodstuff) = 10

#### Variables affecting the marginal productivity of cooking effort

Items in Table 3 are the variables affecting the marginal productivity of the cooking effort, and these are the elements that constitute the vector ***y*** in Eq. (5). The demographic factors of each home cook are surveyed in the Shoku-MAP. Each personality factor in Table 3 is also associated with several questions of the awareness survey in the Shoku-MAP. In the awareness survey, there are multiple yes/no questions asking about each personality factor shown in Table 3. For example, in relation to the “cooking is troublesome” factor, there are questions that ask “yes” or “no” for five different kinds of troublesome. We counted the number of “yes” answers given by each home cook to these five questions, and we defined that the individual falls under the factor “cooking is troublesome” if the number of “yes” answers is higher than the average number of “yes” answers.

**Table 3:** Variables affecting the marginal productivity of cooking effort

Demographic factor	Household income (five levels from < 4 million yen to > 10 million yen)
	Home cook’s age (five levels from 20s to 60s)
	Employment status of home cook (full-time homemaker/full-time worker)
	Living together with or without a family of elderly people (over 60 years old)
Personality factor (awareness information from Shoku-MAP)	Busy
	Cooking is troublesome
	I like eating out
	Buying food is troublesome
	Buying food with emphasis on the price
	Flexible with regard to cooking
	High interest in health
	Prefer luxury foods
	Make plan and cook
	Resistance to purchase cooked food
	Emphasis on the number of foodstuffs
	High interest in diet
	Prefer natural foodstuffs
	Emphasis on the number of dishes
	I like cooking
	Cook on a case-by-case basis

### Estimation formula

When we apply the variables described in the “Creation of indicators” section to Eq. (5), our estimation formula is as follows:

9 $$ {E}_{jn}^{dinner}={\alpha}^{dinner}{F}_{jn}^{dinner}+\sum \limits_i^{16}{\beta}_i^{dinner}\bullet {Person}_{ij}+\sum \limits_k^{10}{\gamma}_k^{dinner}\bullet {Demo}_{kj}+{\delta}^{dinner}+{u}_{jn}^{dinner}. $$

Here, *Person*_*ij*_ is a dummy variable that denotes the *i*th (*i* = 1, ..., 16) personality factor of the *j*th individual. The 16 personality factors are shown in Table 3. *Demo*_*kj*_ denotes the *k*th (*k* = 1, ..., 14) demographic factor of the *j*th individual. The ten demographic factors are a full-time home cook dummy; 4 income class dummies when an annual income of < 4 million yen is the measurement standard (4–6 million yen, 6–8 million yen, 8–10 million yen, and > 10 million); 4 age class dummies, with age in the 20s as the measurement standard (30s, 40s, 50s, and 60s); and a dummy of elderly people (≥ 60 years old) living together. *δ*^dinner^ is a constant term, and ${u}_{jn}^{dinner}$ is an error term. Regression of Eq. (9) was implemented by pooling all 1-year data. We also made similar estimations for breakfast and lunch.

Using the estimate ${\hat{\alpha}}^{dinner}$ of *α*^dinner^ in Eq. (9), we estimated the elasticity ${\hat{\varepsilon}}_{ik}^{dinner}$of the cooking effort with respect to the usage intensity of convenience food for people with the *i*th personality factor and *k*th demographic factor in Eq. (6) as follows:

10 $$ {\hat{\varepsilon}}_{ik}^{dinner}=-{\hat{\alpha}}^{dinner}/\frac{{\overline{E}}_{ik}^{dinner}}{{\overline{F}}_{ik}^{dinner}}. $$

Here, ${\overline{E}}_{ik}^{dinner}$ and ${\overline{F}}_{ik}^{dinner}$ are the average values of the indicators for cooking effort and the usage intensity of convenience food for the person with the *i*th personality factor and *k*th demographic factor, respectively. The same estimation was made for breakfast and lunch.

## Results

### Descriptive analysis

Table 4 contains descriptive statistics of the indicators of cooking effort and usage intensity of convenience food defined in the previous section. The average value of the cooking effort indicator decreased in the order of dinner, breakfast, and lunch, and the average value of the convenience food usage intensity indicator was in the reverse order, suggesting that there may be a negative correlation between cooking effort and the usage intensity of convenience food. This implies that our theoretical model (Eq. (5)) is valid.

**Table 4:** Descriptive statistics of indicators

		Breakfast	Lunch	Dinner
Indicator of the cooking effort (*E*)	Mean	51.413	21.367	95.743
	Std. Dev.	55.071	28.988	83.158
	Max	1406	1060	1701
Indicator of the usage intensity of convenience food (*F*)	Mean	6.797	6.994	4.965
	Std. Dev.	1.728	2.343	1.710
	Max	13	13	13

**Fig. 1:**
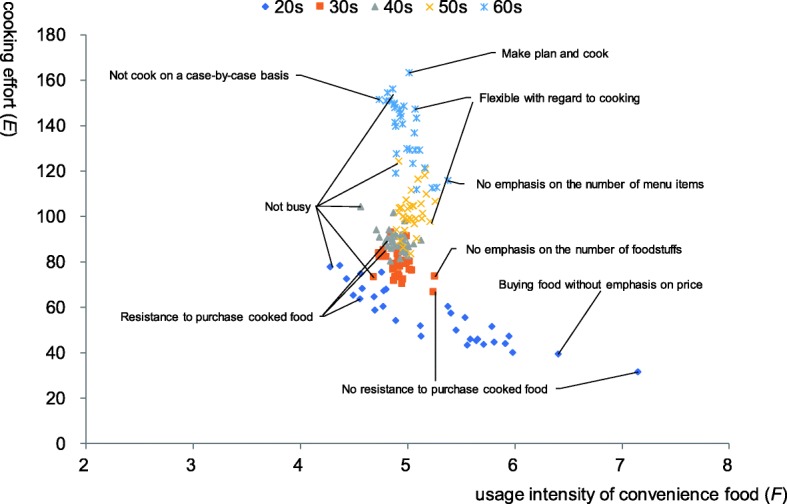
Distribution of the average values of indicators of people with each personality factor by age class

We classified all home cooks by their age and according to the 16 personality factors in Table 3 and calculated the average values of our two indicators for the meals of people belonging to each attribute. The results are plotted in Fig. 1. Cooking effort indicator is higher in households of older home cooks. In younger households, a clearer negative correlation is observed between the level of cooking effort and usage intensity of convenience food, depending on the difference in the home cooks’ personality factors. On the other hand, for older people, such a clear negative correlation is not seen between different attributes. They seem to simply show different cooking efforts for the same convenience food usage, depending on their attributes. The cooking effort indicator is high for people who plan and cook meals, but the indicator is low for people who are not averse to purchasing cooked food. The usage intensity indicator for convenience food is high in people who do not emphasize the number of dishes or foodstuffs, but it is low in people who are not as busy. From these facts, it can be inferred that age and personality factors affect the in-home production of meals of the home cooks. In other words, each home cook chooses a specific amount of cooking effort under a given usage intensity of convenience food according to his/her characteristics of age and personality. The above observations are the result of aggregate values by home cooks’ age and personality factors. In the next section, a more detailed analysis will be performed by regression analysis, using individual sample data.

### Regression analysis

**Fig. 2:**
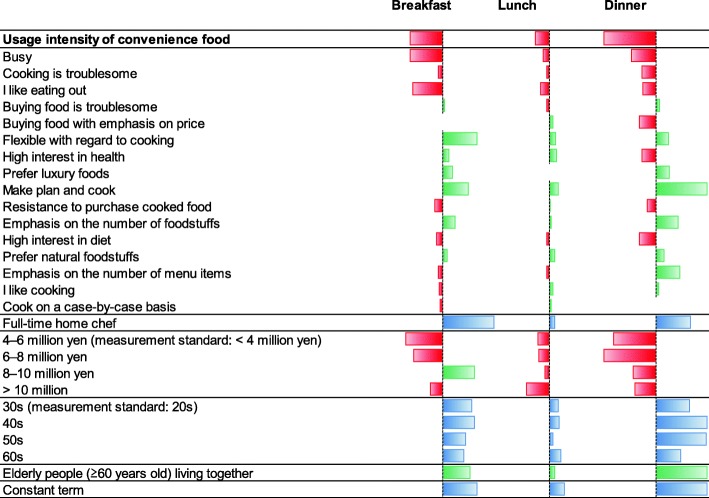
Significant results of the regression analysis. Bars extending to the right indicate positive values, and bars extending to the left indicate negative values

To understand the influence of demographic and personality factors on home production of a home cooks’ meals, we estimated Eq. (9) separately for breakfast, lunch, and dinner in the tobit model. Detailed estimation results are shown in Table 5 in the Appendix. In Fig. 2, we extracted and visualized the significant results in Table 5 in the Appendix. According to Fig. 2, there is a clear negative correlation between the indicators of cooking effort and usage intensity of convenience food in every meal, particularly for dinner. This is consistent with the theory that negative coefficients were estimated for the usage intensity of convenience food for all meals.

People who are flexible with regard to cooking, prefer luxury foods, and plan and cook meals, are concerned with the number of unique foodstuffs, and/or prefer natural foodstuffs have a higher cooking effort indicator under the given indicator of the usage intensity of convenience food. The value is lower for people who are busy, find cooking troublesome, enjoy eating out, and/or have a high interest in their diet. For people who are old or have elderly family members, the value is higher, and for those with middle income, the value is lower. Constant terms are higher in the order of dinner, breakfast, and lunch. This indicates that the average cooking effort indicator for dinner is high.

**Fig. 3:**
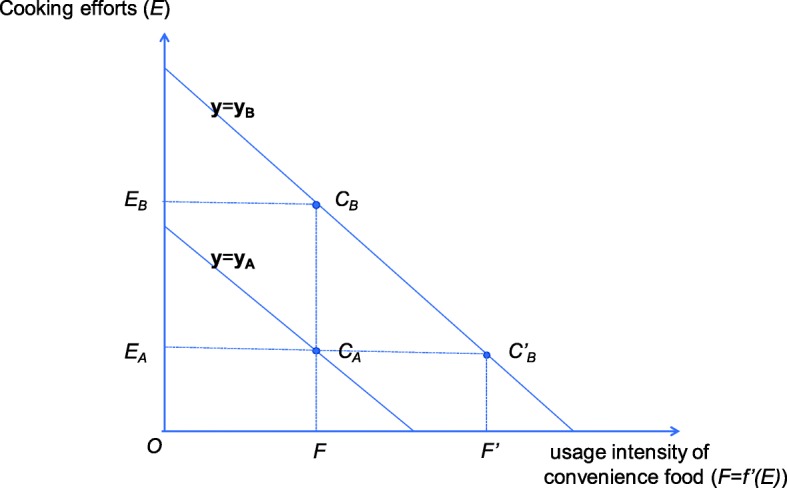
Relationship between cooking effort and usage intensity of convenience food

Home cooks with factors that increase cooking effort indicators can be interpreted as tending to make more of an effort than those without those factors because they are not satisfied with the quality of meals with a given usage intensity of convenience food. This situation is illustrated in Fig. 3.

Figure 3 shows the relationship between the optimal cooking effort and usage intensity of convenience food for two home cooks with attributes ***y***_**A**_ and ***y***_**B**_ in Eq. (5) (e.g., for Mr. A and Mr. B, respectively). On each line, each home cook chooses the optimal cooking effort to apply for a given usage intensity of convenience food. Mr. B’s line is drawn above that of Mr. A. This indicates that Mr. B chooses to apply more cooking effort than Mr. A for the same usage intensity of convenience food. Suppose that the two home cooks eat a piece of bread bought from a food market for breakfast. In Fig. 3, the usage intensity of convenience food of $\overline{OF}$ is assumed for bread. Mr. A with attribute ***y***_**A**_, who values saving time, puts the bread on the table as is, whereas Mr. B with attribute ***y***_**B**_ wants to enjoy his meal and toasts the piece of bread. As a result, Mr. A chooses point *C*_A_ and takes the cooking effort of $\overline{OE_A}$, whereas Mr. B chooses point *C*_B_ and takes the cooking effort of $\overline{OE_B}.$ In order to reduce Mr. B’s cooking effort to the same level as Mr. A, Mr. B needs a higher usage intensity of convenience food $\overline{O{F}^{\prime }}$. For example, if a packed ready-made sandwich is available, which corresponds to $\overline{OF^{\prime }}$, then Mr. B will not spend additional cooking effort and settles at *C*′_B_. This indicates that Mr. B tends to spend more cooking effort or more usage intensity of convenience food than Mr. A for breakfast. Home cooks with attributes that show positive effects in Fig. 2 are those who have such a tendency. Casini et al. [30] found a difference in consumers’ willingness to pay to save cooking time, and they considered the consumer segment important. In that context, Mr. A and Mr. B are consumers belonging to two different segments. The proposed model can explain the background of the facts identified by Casini et al. [30].

In Fig. 2, we pay special attention to the fact that “high interest in diet” is a factor that lowers the cooking effort indicator. Home cooks with this factor continuously gather information about food. We interpret this to mean that home cooks who obtain access to their own convenience food by gathering information effectively reduce their cooking effort. For example, suppose Mr. B uses his smartphone to easily search for information and finds that there is a bakery of freshly baked goods next to the usual food market. Mr. B may think that the bread from the bakery can be served on the table as is. In that case, Mr. B’s line in Fig. 2 is considered to shift downward to the level of Mr. A’s line. ICT-embedded urban systems that use a digital information platform will greatly contribute to providing such information, in a way that is useful to people’s lives [27]. The elderly with cognitive impairments also try to use the search functions of smartphones when they meet their needs in daily activities [28]. It is desirable for home cooks, regardless of their attributes, to be able to reduce their cooking effort by receiving appropriate information through smart food systems using ICT. Smart food systems should be designed to make this possible.

### Estimated results of elasticities of cooking effort with respect to usage intensity of convenience food

**Fig. 4:**
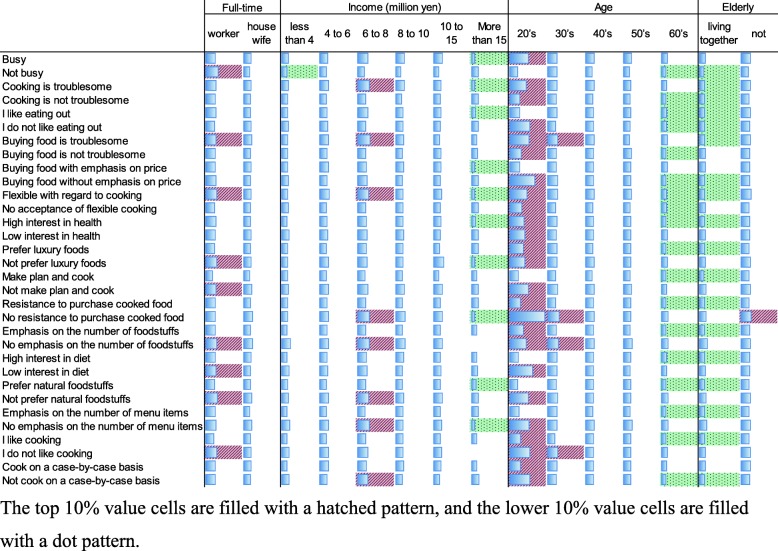
Estimated results of elasticities of cooking effort with respect to usage intensity of convenience food

The elasticities of cooking effort with respect to the usage intensity of convenience food calculated from Eq. (5) or (10) as the empirical form, according to home cooks’ personality and demographic factors, are listed in Table 6 of the Appendix. Figure 4 visually illustrates the same result. For example, the number in the first row of the first column in Fig. 4 shows that as the usage intensity of convenience food increases by 1%, the cooking effort decreases by 0.672% in the meals of home cooks who are “busy” and “full-time workers”. This index represents the reaction speed that home cooks show in terms of cooking effort input for changes in the usage intensity of convenience food.

In Eq. (5) or (10), we define the ratio of changes in cooking effort and changes in usage intensity of convenience food as the same for all home cooks. However, the elasticities in Fig. 4 indicate that the same change has different effects on home cooks with different attributes. Figure 4 shows that home cooks who are employed full-time, with middle-class household income (6–8 million yen), and/or are in their 20s have high elasticities, and home cooks who are older, have elderly family members, and/or have the highest household income (> 15 million yen) have low elasticities. Home cooks who feel that buying food is troublesome, are flexible with regard to cooking, do not prefer luxury foods, do not make plans before cooking, do not show resistance to purchase cooked food, do not emphasize on the number of foodstuffs, are not interested in diet, do not prefer natural foodstuffs, do not emphasize on the number of dishes, and/or do not like cooking have high elasticities. Whereas, home cooks with the opposite personality factors have low elasticities. Here, it is noteworthy that the factor “not interested in diet” is one that increases the elasticity. This means that home cooks who are not interested in diet and probably do not wish to gather food information are more responsive to convenience food usage. Conversely, home cooks who are interested in diet are slow to respond. In the previous section, we pointed out that “interest in diet” is a factor that reduces cooking effort. However, home cooks who are interested in diet seem to be slow to actually reduce cooking effort. This can be interpreted to mean they are cautious about using convenience food because they are particular about diet.

When the usage of convenience food increases in a society where smart food systems have penetrated, people with high elasticity respond sensitively to the change, while people with low elasticity do not. It is desirable that all people to equally benefit from innovation, regardless of their attributes. Home cooks with low elasticity values in Fig. 4 will not be sensitive to more sophisticated convenience food that will be offered under smart food systems. Figure 4 also shows that elderly home cooks or home cooks with elderly families are such people. According to Eq. (5) or (10), it is necessary to decrease $$ \raisebox{1ex}{$E$}\!\left/ \!\raisebox{-1ex}{$F$}\right. $$ in order to increase the elasticity *ε*. Referring to Fig. 3, moving from *C*_B_ to *C*_A_ or *C*′_B_ reduces $$ \raisebox{1ex}{$E$}\!\left/ \!\raisebox{-1ex}{$F$}\right. $$. At present, elderly home cooks are applying a lot of cooking effort for a given usage intensity of convenience food. In order to reduce their $$ \raisebox{1ex}{$E$}\!\left/ \!\raisebox{-1ex}{$F$}\right. $$, they will have to either move to the lower line (move to *C*_A_) or increase their usage intensity of convenience food (move to *C*′_B_). The former change is a downward shift of the meal production function expressed by Eq. (1). The latter change is an expansion of the usage intensity of convenience food. It is desirable that the $$ \raisebox{1ex}{$E$}\!\left/ \!\raisebox{-1ex}{$F$}\right. $$ decreases under a given usage intensity of convenience food (i.e., changes to *C*_*A*_). As discussed in the previous section, under smart food systems, the meal production function of elderly home cooks will shift toward time-saving efforts if convenience food is provided along with appropriate information. The elderly’s $$ \raisebox{1ex}{$E$}\!\left/ \!\raisebox{-1ex}{$F$}\right. $$ will decrease if convenience food that meets their preferences is provided under a well-planned system. The results described in Fig. 4 may lead to interesting suggestions related to the problems that the smart food system should solve.

## Discussion

In this research, we analyzed the Shoku-MAP data [49] provided by Lifescape Marketing Co., Ltd. to assess the implications of a smart food system construction. We defined indicators of cooking effort and usage intensity of convenience food, and presented a model wherein a “meal” is home-produced. In the model, the home cook decides the optimal cooking effort when using a given level of convenience food.

Using an empirical form of the proposed model, we performed a multiple regression analysis using the cooking effort indicator as an explained variable, the indicator of convenience food usage intensity as an explanatory variable, and 16 personality and 14 demographic factors as the shift factor. As a result, significant negative values were estimated for the coefficient of the indicator of convenience food usage intensity. This means that the cooking effort can be compensated by convenience food, which is consistent with our theoretical model of meal production at home. The estimated coefficients for personality and demographic factors showed that a certain level of convenience foods does not result in an equal cooking effort for all home cooks. Factors such as old age and/or the presence of elderly people in the family are increasing the cooking effort. The regression results also showed that “high interest in diet” is a factor that lowers the cooking effort indicator at the given usage intensity of convenience food, suggesting that home cooks who have a high interest in diet and obtain access to their own convenience food by gathering information effectively reduce their cooking effort.

Using the estimated coefficients for the indicator of convenience food usage intensity in the above multiple regression analysis, we calculated “the elasticity of cooking effort with respect to usage intensity of convenience food” for home cooks, with each attribute defined by a combination of different personality and demographic factors. As a result, low elasticities were calculated for home cooks who are old (≥ 60 years old), have elderly people in the family, and/or have special food preferences. Home cooks with low elasticity values will not react sensitively to the provision of more sophisticated convenience food.

These results show that existing convenience foods do not have the same impact on home cooks with different attributes. In particular, we should note that home cooks who are old and/or have elderly in the family apply relatively high cooking effort for a given level of convenience food, and are not sensitive to the increase in convenience food. According to the UN population data, the population percentage of elderly people (≥ 65 years old) in Japan was 27% in 2017, the highest among developed countries. Previous studies [18–20] have demonstrated that the daily diet of the elderly is of nutritional concern. This will soon become a serious problem in Japan. Although the use of convenience food is thought to be useful for nutritionally improving the diets of the elderly, the results of this study suggest that it seems difficult for the elderly to accept convenience food in its current form.

The role of information for consumers to access healthy food is thought to be important [26–29]. If the elderly can properly obtain information on stores selling convenience foods that are most suitable for them, they may be able to accept convenience food as it is without the need for extra cooking effort. Further, if food suppliers can properly manage information about the preferences of the elderly, they may be able to provide diets that the elderly truly desire and contribute toward reducing their cooking effort and improving nutrition. The same situation may apply to home cooks who are full-time workers and pay particular attention to the quality of their diet. A smart food system that utilizes ICT and that allows home cooks to explore appropriate food information will enable home cooks with any attributes to obtain equal utility without additional cooking effort by themselves for a given usage intensity of convenience food. Our regression analysis shows that “high interest in diet” is a factor of reduced cooking effort for the same usage intensity of convenience food. It seems that home cooks who have a high interest in diet gather more information about food, a fact that strengthens our hypothesis. We must improve the accuracy of this hypothesis in future investigations.

The limitation of this study is the lack of evidence on the relationship between the expansion of information presented by ICT and a change in the way home cooks buy their food. What kind of information about eating habits do home cooks get from smartphones and social network services? How are cooking and food shopping behaviors of the home cooks changing by utilizing this information? In the future, we will conduct a large-scale questionnaire survey for home cooks to investigate these questions. The result will be useful for the construction of a smart food system that allows any home cook to cook convenient and nutritionally rich meals. As another limitation, this study excludes single-person household data because sufficient information on eating habits awareness was not available for such households. Research on single-person households is our future task.

## Conclusions

In recent years, the evaluation of convenience food has changed. It came to be considered not to have a negative effect on health and is now positioned as a tool to support the dietary habits of the elderly and other people. Furthermore, in developed countries where the population is aging, convenience foods are expected to improve the eating habits of the elderly. We applied a home production function model and constructed a model that explains the behavior of home cooks replacing cooking effort with convenience food. Unlike other compulsory efforts, such as commuting, the amount of distress brought by the cooking effort varies greatly depending on individual attributes. As a result, depending on the individual attributes, the way of using convenience food varies greatly. Empirical analysis shows that older people are reluctant to replace their cooking effort with convenience food. Our empirical results also suggest that those who gather information are appropriately replacing their cooking efforts with convenience food. Based on the results, a hypothesis is derived that it is effective to provide information using smartphones to encourage the elderly to use convenience foods appropriately. Our future research topic is to verify this hypothesis empirically.

## Data Availability

Data sharing is not applicable to this article as no datasets were generated or analyzed during the current study.

## Appendix

**Table 5:** Results of regression analysis: determinants of the cooking effort indicator (*E*)

	Breakfast	Lunch	Dinner
Usage intensity of convenience food (*F*)	− 6.751 (0.093)***	− 2.884 (0.049)***	− 10.662 (0.125)***
Busy	− 13.902 (0.535)***	− 2.530 (0.334)***	− 10.458 (0.833)***
Cooking is troublesome	− 1.885 (0.405)***	− 1.111 (0.332)***	− 6.037 (0.672)***
I like eating out	− 12.568 (0.400)***	− 3.741 (0.300)***	− 5.416 (0.630)***
Buying food is troublesome	0.891 (0.459)*	− 0.978 (0.323)***	1.440 (0.693)**
Buying food with emphasis on price	− 0.328 (0.410)	1.560 (0.290)***	− 7.260 (0.667)***
Flexible with regard to cooking	14.548 (0.403)***	2.688 (0.285)***	5.288 (0.626)***
High interest in health	2.776 (0.394)***	2.819 (0.336)***	− 5.905 (0.669)***
Prefer luxury foods	3.953 (0.370)***	0.025 (0.316)	5.744 (0.657)***
Make plan and cook	10.915 (0.380)***	3.677 (0.267)***	21.980 (0.658)***
Resistance to purchase cooked food	− 3.169 (0.425)***	0.538 (0.272)**	− 3.759 (0.678)***
Emphasis on the number of foodstuffs	5.214 (0.424)***	0.656 (0.291)**	9.266 (0.651)***
High interest in diet	− 2.624 (0.403)***	− 1.009 (0.306)***	− 7.068 (0.684)***
Prefer natural foodstuffs	1.821 (0.402)***	2.141 (0.327)***	3.503 (0.697)***
Emphasis on the number of dishes	− 1.744 (0.434)***	− 0.977 (0.319)***	10.205 (0.710)***
I like cooking	− 1.436 (0.422)***	1.542 (0.357)***	1.214 (0.715)*
Cook on a case-by-case basis	− 0.995 (0.379)***	0.870 (0.288)***	− 0.878 (0.622)
Full-time home cook	15.206 (0.371)***	1.475 (0.262)***	10.144 (0.603)***
4–6 million yen (measurement standard < 4 million yen)	− 13.326 (0.719)***	− 4.166 (0.453)***	− 15.041 (1.169)***
6–8 million yen	− 10.463 (0.718)***	− 3.910 (0.487)***	− 18.575 (1.175)***
8–10 million yen	11.378 (0.978)***	− 1.463 (0.545)***	− 8.275 (1.214)***
>10 million	− 4.297 (0.807)***	− 8.190 (0.525)***	− 7.602 (1.310)***
30s (measurement standard, 20s)	13.718 (0.592)***	3.963 (0.648)***	15.997 (1.016)***
40s	15.051 (0.596)***	4.554 (0.614)***	24.588 (0.988)***
50s	10.642 (0.643)***	1.624 (0.685)**	23.915 (1.107)***
60s	10.107 (1.362)***	5.420 (0.998)***	11.523 (2.153)***
Elderly people (≥ 60 years old) living together	28.266 (0.987)***	5.442 (0.627)***	53.760 (1.575)***
Constant term	81.262 (1.218)***	36.284 (0.811)***	123.365 (1.640)***
Log-likelihood	− 405,439.480	− 222,594.860	− 401,985.520
Pseudo *R*^2^	0.023	0.012	0.019
Number of obs	76,444	47,063	70,151

Numbers in parentheses indicate robust standard errors

***, **, and * indicate that the estimate is statistically significant with a significance level of 1%, 5%, and 10%, respectively

**Table 6:** Elasticities of cooking effort (*E*) with respect to usage intensity of convenience food (*F*)

Demographic factor															
	Full-time		Income (million yen)						Age					Elderly	
Personality factor	Worker	Home cook	<4	4–6	6–8	8–10	10–15	> 15	20s	30s	40s	50s	60s	Living together	Not living together
Busy	0.672	0.559	0.492	0.607	0.712	0.613	0.631	*0.251*	**1.313**	0.661	0.617	0.603	0.415	0.411	0.684
Not busy	**0.859**	0.427	*0.374*	0.466	0.501	0.391	0.397	0.483	0.588	0.684	0.467	0.422	*0.332*	*0.328*	0.522
Cooking is troublesome	0.708	0.554	0.478	0.645	**0.810**	0.629	0.521	*0.251*	**1.169**	0.679	0.633	0.553	0.410	*0.374*	0.706
Cooking is not troublesome	0.683	0.486	0.433	0.498	0.569	0.456	0.506	0.483	**0.755**	0.646	0.547	0.507	*0.361*	*0.372*	0.582
I like eating out	0.726	0.507	0.428	0.551	0.715	0.554	0.532	*0.312*	0.717	0.705	0.596	0.560	*0.376*	*0.370*	0.663
I do not like eating out	0.634	0.525	0.486	0.585	0.582	0.519	0.478	0.494	**1.395**	0.609	0.576	0.496	*0.366*	*0.376*	0.618
Buying food is troublesome	**0.752**	0.568	0.421	0.646	**0.881**	0.584	0.441	0.406	**1.341**	**0.755**	0.646	0.458	0.437	*0.349*	0.705
Buying food is not troublesome	0.670	0.497	0.463	0.529	0.604	0.510	0.526	0.441	**0.846**	0.629	0.563	0.567	*0.362*	0.379	0.616
Buying food with emphasis on price	0.674	0.530	0.416	0.626	0.640	0.577	0.535	*0.251*	0.735	0.629	0.568	0.547	0.418	0.381	0.636
Buying food without emphasis on price	0.707	0.498	0.497	0.519	0.684	0.510	0.493	0.483	**1.739**	0.728	0.606	0.516	*0.349*	*0.367*	0.650
Flexible with regard to cooking	**0.788**	0.540	0.414	0.670	**0.764**	0.554	0.603	*0.251*	**1.205**	0.707	0.615	0.569	*0.368*	*0.361*	0.709
Not flexible with regard to cooking	0.619	0.491	0.499	0.476	0.571	0.527	0.472	0.483	**0.856**	0.604	0.557	0.507	*0.374*	0.380	0.582
High interest in health	0.708	0.479	0.386	0.546	0.641	0.512	0.524	*0.312*	**1.007**	0.681	0.603	0.541	*0.357*	*0.361*	0.651
Low interest in health	0.683	0.555	0.531	0.584	0.679	0.580	0.494	0.494	**1.053**	0.657	0.574	0.517	0.438	0.399	0.637
Prefer luxury foods	0.639	0.490	0.444	0.482	0.653	0.545	0.461	0.483	**0.969**	0.671	0.542	0.546	*0.342*	*0.367*	0.606
Not prefer luxury foods	**0.782**	0.540	0.459	0.647	0.667	0.529	0.728	*0.251*	**1.068**	0.661	0.643	0.514	0.422	0.381	0.675
Make plan and cook	0.468	0.430	0.425	0.506	0.533	0.473	0.403	0.383	0.651	0.585	0.511	0.466	*0.328*	*0.320*	0.565
Not make plan and cook	**0.748**	0.603	0.476	0.611	0.729	0.612	0.646	0.482	**1.299**	0.713	0.646	0.591	0.410	0.428	0.698
Resistance to purchase cooked food	0.705	0.489	0.450	0.527	0.598	0.499	0.490	0.483	**0.763**	0.602	0.575	0.530	*0.333*	*0.355*	0.601
No resistance to purchase cooked food	0.684	0.613	0.463	0.649	**0.807**	0.671	0.649	*0.251*	**2.436**	**0.841**	0.611	0.526	0.498	0.425	**0.751**
Emphasis on the number of foodstuffs	0.676	0.477	0.377	0.544	0.605	0.474	0.476	0.411	**0.952**	0.618	0.563	0.481	*0.357*	*0.347*	0.609
No emphasis on the number of foodstuffs	**0.761**	0.622	0.643	0.616	**0.817**	0.662	0.624		**1.159**	**0.762**	0.624	0.643	0.485	0.512	0.704
High interest in diet	0.663	0.525	0.388	0.548	0.642	0.571	0.510	0.406	0.653	0.672	0.603	0.556	*0.368*	*0.351*	0.641
Low interest in diet	**0.745**	0.503	0.521	0.583	0.687	0.507	0.515	0.441	**1.604**	0.658	0.575	0.503	0.378	0.384	0.645
Prefer natural foodstuffs	0.630	0.472	0.429	0.501	0.575	0.504	0.488	*0.361*	0.596	0.613	0.582	0.536	*0.347*	*0.339*	0.604
Not prefer natural foodstuffs	**0.845**	0.576	0.483	0.642	**0.779**	0.599	0.627	0.484	**1.336**	0.714	0.592	0.518	0.496	0.434	0.687
Emphasis on the number of dishes	0.664	0.451	0.380	0.487	0.532	0.514	0.465	0.483	0.672	0.670	0.535	0.467	*0.339*	*0.339*	0.597
No emphasis on the number of dishes	0.724	0.581	0.570	0.624	**0.827**	0.559	0.593	*0.251*	**1.378**	0.661	0.617	0.613	0.495	0.474	0.676
I like cooking	0.606	0.491	0.383	0.489	0.593	0.535	0.471	0.411	**0.775**	0.559	0.592	0.516	*0.352*	*0.359*	0.592
I do not like cooking	**0.794**	0.540	0.551	0.652	0.733	0.540	0.571		**1.434**	**0.736**	0.583	0.543	0.454	0.404	0.686
Cook on a case-by-case basis	0.698	0.528	0.414	0.613	0.615	0.589	0.558	0.393	**0.761**	0.701	0.596	0.539	0.396	0.386	0.660
Not cook on a case-by-case basis	0.693	0.499	0.522	0.519	**0.737**	0.477	0.454	0.526	**1.396**	0.625	0.575	0.515	*0.333*	*0.349*	0.623

The top 10% value cells are shown in bold. The lower 10% value cells are shown in italics

## Abbreviations

Shoku-MAP: Shokutaku (Table) Market Analysis and Planning
ICT: Information and communication technology
